# Enantioselective Cytotoxicity Profile of *o,p*’-DDT in PC 12 Cells

**DOI:** 10.1371/journal.pone.0043823

**Published:** 2012-08-24

**Authors:** Meirong Zhao, Cui Wang, Chunlong Zhang, Yuezhong Wen, Weiping Liu

**Affiliations:** 1 Research Center of Environmental Science, Zhejiang University of Technology, Hangzhou, China; 2 University of Houston-Clear Lake, Houston, Texas, United States of America; 3 Institute of Environmental Sciences, College of Environmental and Resource Sciences, Zhejiang University, Hangzhou, China; Indian Institute of Toxicology Research, India

## Abstract

**Background:**

The continued uses of dichlordiphenyltrichloroethane (DDT) for indoor vector control in some developing countries have recently fueled intensive debates toward the global ban of this persistent legacy contaminant. Current approaches for ecological and health risk assessment has ignored the chiral nature of DDT. In this study by employing an array of cytotoxicity related endpoints, we investigated the enantioselective cytotoxicity of *o,p*’-DDT.

**Principal Findings:**

we demonstrated for the first time that *R*-(−)-*o,p*’-DDT caused more neuron cell death by inducing more severe oxidative stress, which selectively imbalanced the transcription of stress-related genes (SOD1, SOD2, HSP70) and enzyme (superoxide dismutase and lactate dehydrogenase) activities, and greater cellular apoptosis compared to its enantiomer *S*-(+)-*o,p*’-DDT at the level comparable to malaria area exposure (parts per million). We further elucidated enantioselective modes of action using microarray combined with enzyme-linked immunosorbent assay. The enantioselective apoptosis might involve three signaling pathways via caspase 3, tumor protein 53 (p53) and NF_k_B.

**Conclusions:**

Based on DDT stereochemistry and results reported for other chiral pesticides, our results pointed to the same directional enantioselectivity of chiral DDT toward mammalian cells. We proposed that risk assessment on DDT should consider the enantiomer ratio and enantioselectivities.

## Introduction

Dichlordiphenyltrichloroethane (DDT) was hailed as an effective insecticide to control malaria and typhus in 1940s. It was banned for agricultural use in 1970s–1980s primarily on the basis of ecological consideration [1]. When DDT emissions ceased in 1990, about 634 kt DDT were released into the environment [2]. Even though the Stockholm Convention on Persistent Organic Pollutants listed DDT as the “Dirty Dozen" in 2001 for the global community [3], DDT is still currently used in indoor residue spraying (IRS) in 14 tropical countries and several other countries are preparing to reintroduce it [4]. High levels of DDT (parts per million levels) were always detected in malaria control area. Taken IRS areas of South Africa for example, the mean DDT concentration approached 7.3 µg/g in human serum and 240 mg/kg in chicken fat [5]. Because of the nature of high bioaccumulation, DDT level in organism would be elevated in the future in malaria control area. As DDT has gained renewed interest, the debates continue regarding its benefits and risks.

Several decades of studies on DDT have given its notoriety for egg shell thinning, intersex, neurological and carcinogenic effects [6]. However, these studies have mainly originated in developed countries with lower levels of DDT than that in malaria control areas [7], [8]. As a potential neurotoxic pesticide, DDT preferentially accumulates (one hundred times greater) in organs containing a high level of lipid such as neural system [9]. Neuron injuries or neurodegenerate diseases have been reported at high doses of occupational or indoor direct exposure [10], [11]. DDT-induced neuronal cell death has also been concerned recently with limited studies using *in vitro* model [12]–[14], whereas the molecular mechanism for DDT-induced cellular toxicity remains widely unknown.

Complications arise for the risk assessment of DDTs because one of the steroisomers *o*,*p*’-DDT is chiral. Technical DDT comprises approximately 75% achiral *p,p*’-DDT and only 25% chiral *o,p*’-DDT [15]. However, in some area such as the Taihu Lake of China, the average concentration of *o,p*’-DDT was reportedly 6.3 times higher than p,p’-DDT in the air [16], which indicated this minor component cannot be neglected. Since *R*-(−)-enantiomer is preferentially metabolized in animals, *S*-(+)-*o,p*’-DDT would largely accumulate [15], [17]. Consequently, risk assessment based on enantioselective toxicity would be much more accurate than that using its racemate. Our group has recently clarified the enantioselective toxicity of several chiral pesticides and demonstrated the enantioselective endocrine disruption, immunotoxicity, cytotoxicity as well as development toxicity [18]–[23]. For *o,p*’-DDT, our result [19] was in accordance with other chiral pesticides in that the *R*-enantiomer is the more active estrogen mimic [24]–[27]. However, concerns to date regarding the enantioselective environmental safety and health risk of o,*p*’-DDT have only been focused on estrogenic activities, the enantioselective toxicity (particularly neurocyte toxicity and mechanism) associated with *o,p*’-DDT [28] has never been reported.

Herein we conducted a systematic study on *o,p*’-DDT induced stereoselective cytotoxicity by exploring a widely used *in vitro* neuron cell model with PC12 cells. A plausible molecular mechanism is also proposed, for the first time, based on microarray analysis. The goal was to determine the extent of enantioselective cytotoxicity of *o,p*’-DDT under the dose at part per million levels typical of malaria control areas. The implications of our results in respect to risk assessment of chiral DDT and other chiral compounds of environmental importance are discussed.

## Results

### Enantioselectivity in Cell Viability

**Figure 1:**
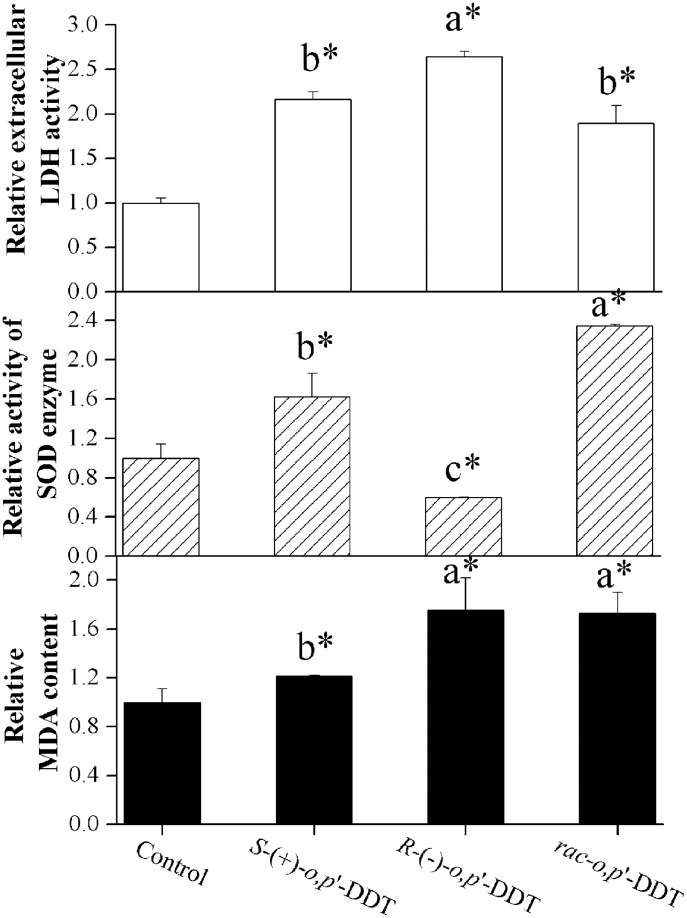
Oxidative stress induced by racemate and enantiomers of *o,p’*-DDT. Effect of the racemate of *o,p’*-DDT and individual stereoisomers on extracellular lactate dehydrogenase (LDH) release, intracellular superoxide dismutase (SOD) and malondialdehyde (MDA) production at concentration of 3.5×10^−5^ mol/L. Data are presented as the relative value of control. PC12 cells were exposed to different compounds for 24 h. The asterisk above each bar indicates a significant difference compared to a negative control (*p*<0.05, n = 3). Different letters above adjacent bars indicate a significant difference (*p*<0.05) between the two enantiomers, whereas the same letter indicates no significant difference.

Cell damage and viability as measured by lactate dehydrogenase (LDH) are shown in **Fig. S1** (**A, B**). LDH activities were significantly increased at a dose dependent manner after treatment with *rac-o,p*’-DDT, a 1.8-fold increase at 3.5×10^−5^ mol/L (*p*<0.05). At this concentration, *R*-(−)-*o,p*’-DDT induced 1014.9 U/L LDH compared with 828.8 U/L of *S*-(+)-*o,p*’-DDT. An enantioselective leakage of LDH (reported in its relative form) triggered by the two enantiomers of *o,p*’-DDT is shown in Fig. 1.

### Enantioselectivity in Oxidative Stress Effect

The increased anti-oxidative enzymatic activities of superoxide dismutase (SOD) and the accumulation of oxidant malondialdehyde (MDA) in PC12 cells in response to oxidative stress are also shown in **Fig. 1**. The spectrophotometric analysis data (**Fig. S2**) showed that PC12 cells exposed to *rac-o,p*’-DDT had a significant increase (2.3-fold) in SOD when compared to the negative control in a dose-dependent manner. The two enantiomers of *o,p*’-DDT, however, displayed obvious enantioselective effect on SOD activity, i. e., *S-*(*+*)*-o,p*’-DDT significantly increased SOD while *R-*(−)*-o,p*’-DDT significantly decreased its activity (*p*<0.05, **Fig. 1**).

For the oxidant MDA production, *rac-o,p*’-DDT significantly induced MDA in PC12 cells, indicating a severe oxidative damage (*p*<0.05). At the enantiomeric level, both enantiomers increased MDA contents expressed in nmol/mg protein. However, *R*-(−)-*o,p*’-DDT induced 1.4-fold more MDA than that of *S*-form (*p*<0.05). The different regulations of oxidative stress genes described below are intended to further examine their association with enantioselective alterations of the activities of both oxidants (MDA) and antioxidant enzyme (SOD).

### Alteration of Gene Expression Encoding Antioxidative Enzymes and Heat Shock Protein

**Figure 2:**
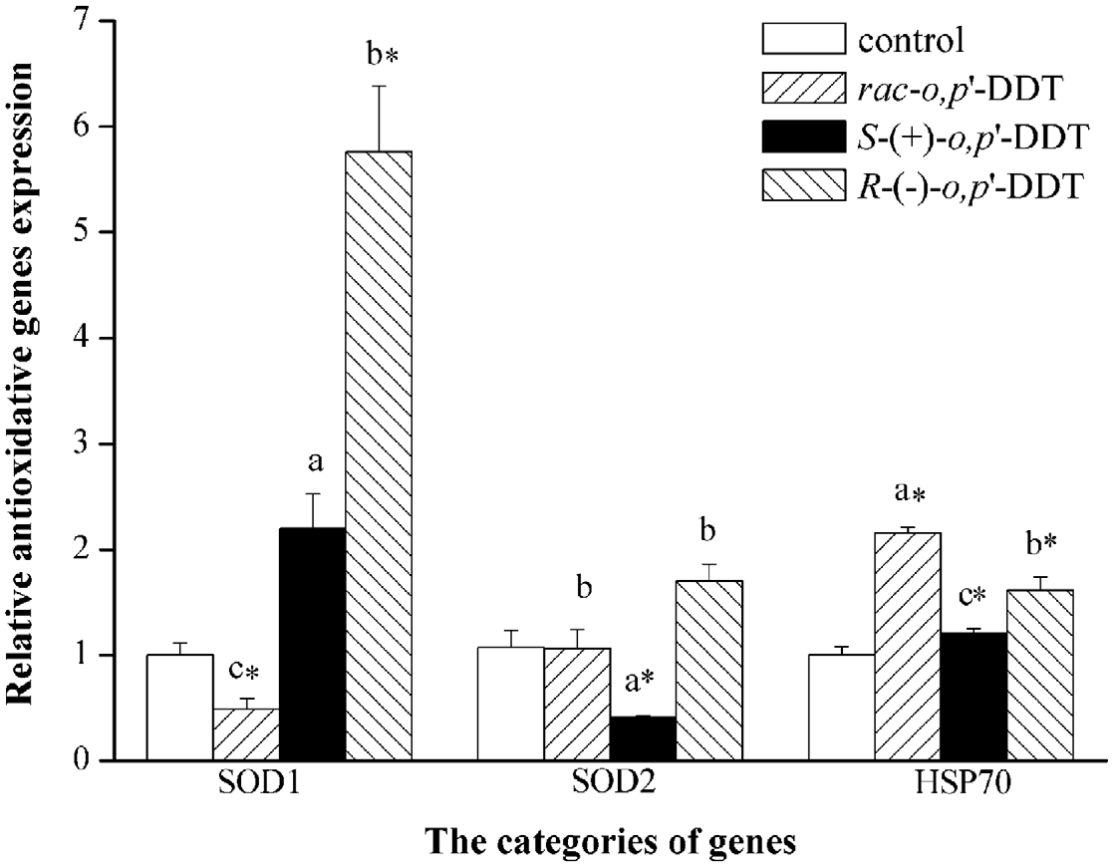
Oxidative stress related gene induction by racemate and enantiomers of *o,p’*-DDT. The relative expression of anti-oxidative related genes encoding superoxide dismutase (SOD1 and SOD2) and heat shock protein (HSP) genes in response to racemate and enantiomers of *o,p*’-DDT. The asterisk denoted *p*<0.05 relative to the negative control. Different letters above adjacent bars indicate a significant difference (*p*<0.05) between individual enantiomers or between an enantiomer and racemate, while the same letter indicates no significant difference.

Since mild oxidative stress together with stress protein induction often result in cell death in vertebrate cells [29], we determined the mRNA levels of antioxidative-related genes (two major isomers encoded SOD, i.e., SOD1, SOD2) as well as a set of stress response genes (**Table S1**) which are induced to synthesize a group of heat shock proteins (HSPs). Exposure to *o,p*’-DDT for 24 h showed a small but significant downregulation of SOD1 rather than SOD2 (**Fig. 2**). Enantioselective transcription has been observed between the two enantiomers. *R-*(−)*-o,p*’-DDT exhibited a significant upregulation of SOD1, while *S-*form had no significant effect (*p* = 0.07) on the expression of anti-oxidative SOD1. Downregulation of SOD2 was observed in the treatment of *S-*(+)-*o,p*’-DDT, compared with the lack of induction by the *R-*form. Among genes encoding the HSPs, only HSP70 was significantly upregulated by *rac-o,p*’-DDT. Furthermore, enantioselective increases in the expression of HSP70 were noted between the two enantiomers with the induction order of *S-*(+)*-o,p*’-DDT < *R*-(−)*-o,p*’-DDT (**Fig. 2**).

### Enantioselectivity in Apoptosis Induction

**Figure 3:**
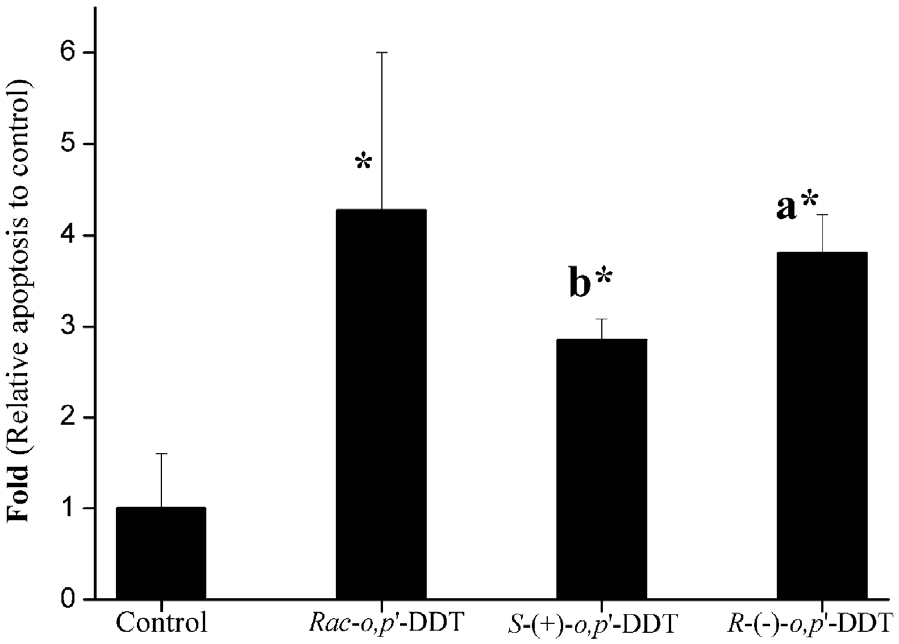
Enantioselectivity in DDT-inducted apoptosis in PC12 cell. The percent of early apoptotic cells following treatment with individual enantiomers or racemate of DDT was measured with flow cytometry quantified by Annexin V (AV)-PI staining.

*rac-o,p’*-DDT and both enantiomers induced apoptosis in PC12 cells after 24 h exposure (see **Fig. 3** for relative data). *rac*-*o,p*’-DDT induced the most significant apoptosis in PC12 cells (14.4%) compared with 2.4% of control group, implying that the racemate is more toxic to the neuron cells. *R*-(−)-*o,p*’-DDT induced 10.3% of cellular apoptosis, while *S*-(+)-*o,p*’-DDT caused only 7.2%, which showed the enantioselective apoptotic induction.

### Enantioselectivity in Altering Apoptosis-related Genes Expression by Microarray and qRT-PCR Validation

**Figure 4:**
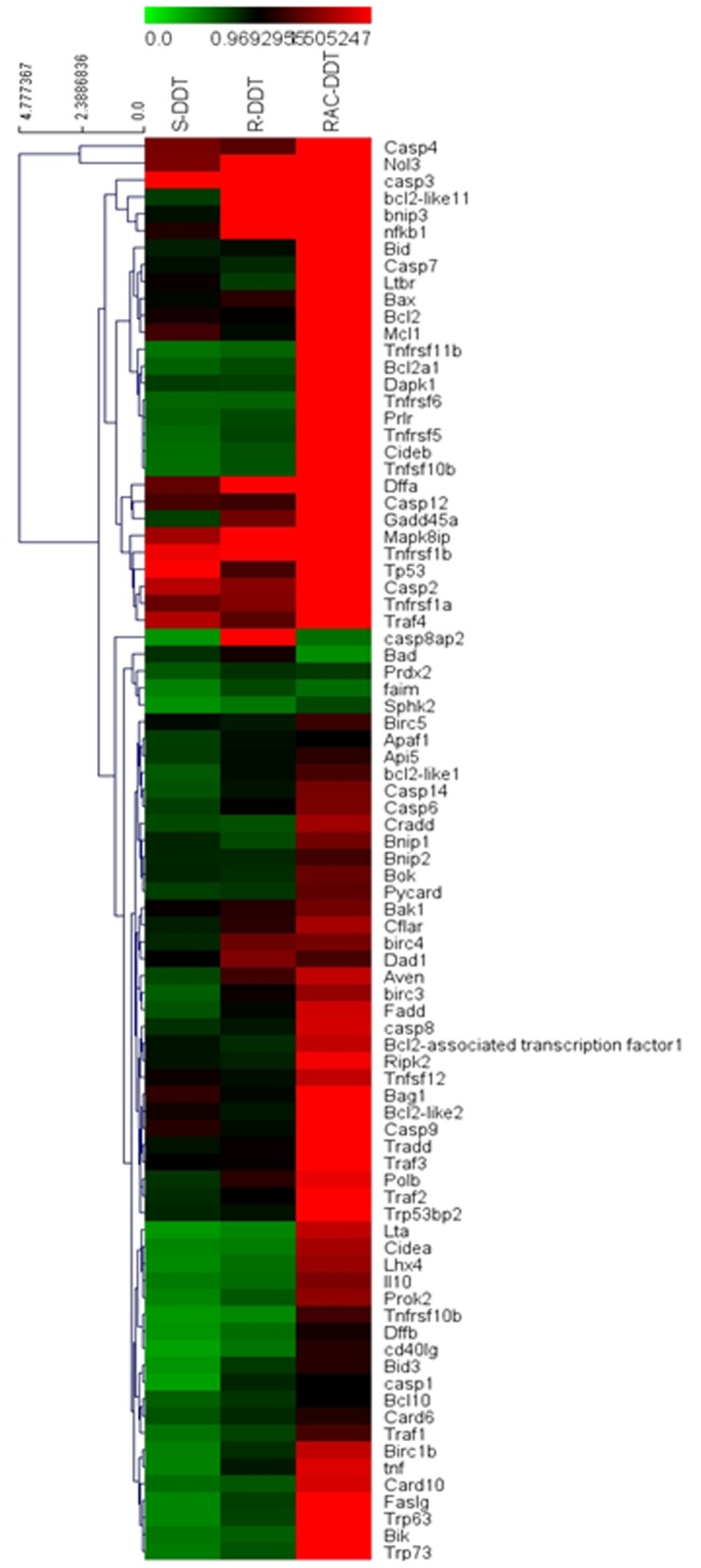
Heatmap of 84 apoptotic genes. The hierarchical clustering was performed for microarray data. Green indicates relative low expression, and red indicates relative high expression.

As can be seen in Fig. 4, approximately 52, 39, and 31 genes, involved in most of families, were changed under the treatment of *rac*-*o,p*’-DDT, *R*-(−)-*o,p*’-DDT and *S*-(+)-*o,p*’-DDT, respectively. Among them, 18 members of genes displayed enantioselecitivity. The disrupted transcription of TNF, caspase, Bcl-2 and p53, all critical to apoptosis, are further described below.

#### TNF family

The most important extrinsic pathway for triggering apoptosis is the TNF family as a remarkable change was noted in gene expression after 24 h *rac-o,p*’-DDT exposure (**Table S2**). The members of TNF ligand family such as TNF, Tnfsf12 and Faslg were upregulated approximately 1.5-fold by *rac*-*o,p*’-DDT. Among TNF receptor family, Ltbr, Tnfrsf11b, Tnfrsf1a and Tnfrsf1b were upregulated 1.9 to 2-fold, whereas Tnfrsf5, Tnfrsf6 and Tnfrsf10 were enhanced 1.7-fold. Between two enantiomers, the mRNA levels of TNF and Faslg under the treatment of *R-*(−)*-o,p*’-DDT was about 1.82 and 1.42-fold higher than that of *S*-(+)-*o,p*’-DDT.

#### Caspase family

Exposure to *rac-o,p*’-DDT increased caspase 1.4–2.7 fold (**Table S3**). The activator caspase 2 and 8 were upregulated 2- and 1.4-fold, respectively. The expression of executioner caspase 3 and 7 were increased about 2-fold. Moreover, caspase 12 commonly found in endoplasmic reticulun was increased 2.7-fold. The inflammatory mediator caspase 4 increased expression almost 7-fold. Exposure to *R*-(−)-*o,p*’-DDT had a greater alteration of the mRNA expression of caspase 1 and 3 compared to *S*-(+)-*o,p*’-DDT. For the regulation of caspase 8ap2 gene expression, *R*-(−)-o,p’-DDT increased the mRNA level 1.8 fold compared to a 2.5-fold downregulation for *S*-(+)-*o,p*’-DDT. The enantioselective induction of caspase 8ap2 by *R*-(−)-o,p’-DDT is approximately 4.5-fold greater than that by the *S*-form.

#### Bcl-2 family

The expressions of 19 genes in Bcl-2 family were analyzed (**Table S4**) given its importance for Bcl-2 related molecules in the apoptosis. Bax and MCl1 were upregulated 1.9-fold in response to *rac*-*o,p*’-DDT, and Bcl-2l1, Bcl-2l2 and the Bcl-2 aderiouvirus E1b19KD interacting protein 3 (Bnip3) were upregulated to approximately 1.6-fold. Only the Bad was downregulated 2.0 fold after exposure to *rac*-*o,p*’-DDT. When compared the expression induced by *R*-(−)-*o,p*’-DDT to that by *S*-(+)-*o,p*’-DDT, a 2.17-, 1.85-, and 2.33-fold difference in the levels of Bcl-2l11, Bid3 and Bnip3 was noted.

#### p53 and DNA damage-induced family

Results with *rac*-*o,p*’-DDT (**Table S5**) indicated that p53 was upregulated 2.3-fold whereas Trp63, Trp73 and Trp53bp2 were increased nearly 1.6-fold. Faim (the apoptotic inhibitory molecule), Prdx2 (an important gene in eliminating peroxides generation regulator) and Sphk2 (a lipid mediator) were downregulated. In contrast, the levels of mRNA of Mapk8ip involved in stress-activated protein kinase (SAPK) mediated stress-induced cellular responses, NF*_k_*B1, polymerase beta (Polb) (important for base excision repair), Prlr (a receptor for the anterior pituitary hormone prolactin) were activated (**Table S6**). As compared to other families, only several genes in p53 and DNA-damage induced apoptosis family exhibited a slight enantioselectivity in transcription.

The disrupted transcriptions of other gene families are detailed in the Supporting Information (**Tables S7, S8, S9, S10, S11**). Overall, for the apoptotic-induced genes the alteration was generally greater when treated with *R*-(−)-*o,p*’-DDT than *S*-(+)-*o,p*’-DDT.

The preceding results based on microarray technique were further validated by RT-PCR, because RT-PCR is more sensitive and capable of reducing the false-positive/negative error. To this end, only a part of genes in TNF, Bcl2, caspase, p53 and some anti-apoptotic families with more than 1.8-fold induction were chosen to do the verification. Results summarized in **Table 1** and **Table 2** clearly shows a good agreement between the two methods, indicating the reliability of enantioselective results obtained from the microarray.

**Table 1:** Results of RT-PCR verification of microarray on *rac-o,p*’-DDT.

Gene name	Gene Bank	*Rac*-*o,p*’-DDT/Control	
		Fold Change by Microarray	Fold Change by Real- time PCR
TNFrsf11b	NM_012870.2	2.0	3.8
TNFrsf1a	NM_013091.1	2.1	1.5
Casp4	NM_053736.2	7.3	6.4
Casp3	NM_012922.2	1.9	1.4
Casp7	NM_022260.2	1.8	2.1
Bid	NM_022684.1	2.3	2.2
Bax	NM_017059.1	1.9	1.6
Dffa	NM_053679.2	3.0	1.4
Gadd45A	NM_024127.2	2.9	4.9
Tp53	NM_030989.1	2.3	1.5
Mapk8ip1	NM_053777.1	2.0	0.9
NF*k*B1	XM_001075876.2	1.8	2.7

**Table 2:** Results of RT-PCR verification of microarray on enantiomers of *o,p*’-DDT.

Gene Name	Gene Bank	Fold Change by Real-time PCR	
		*S*-(+)-*o,p*’-DDT/Control	*R*-(−)-*o,p*’-DDT/Control
TNF	NM_012675.2	1.8	2.7
Casp1	NM_012762.2	6.9	9.8
Casp3	NM_012922.2	1.8	2.8
bcl2l11	NM_022612.1	1.2	2.4
bnip3	NM_053420.2	2.6	2.3
birc1b	XM_001070799.1	1.8	2.6
birc3	NM_023987.2	2.1	1.7
Gadd45a	NM_024127.2	2.9	2.4
Tp53	NM_030989.1	1.4	0.8
NF*k*B	XM_001075876.2	2.5	2.1
Aven	NM_001107757.1	1.5	1.2

### Enantioselectivity of *o,p*’-DDT on the Expression of p53, Caspase 3 and NF*_k_*B

**Figure 5:**
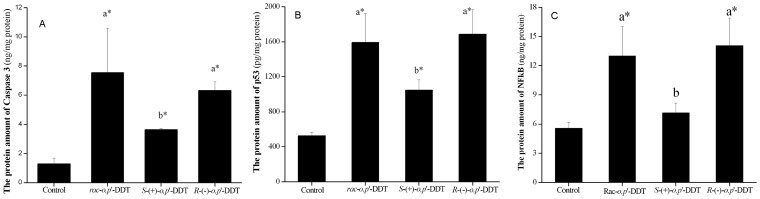
Apoptotic related proteins induced by racemate and enantiomers of o,p’-DDT. The protein expression of (A) p53, (B) caspase3 and (C) NF*_k_*B after exposured to *rac*-*o,p*’-DDT, *R*-(+)-*o,p*’-DDT, and *S*-(−)-*o,p*’-DDT. The asterisk indicates *p*<0.05 relative to a negative control. Different capitalized letters above adjacent bars indicate a significant difference (*p*<0.05) between individual enantiomers or between an enantiomer and racemate, while the same letter indicates no significant difference.

Apart from gene expression results, three important apoptotic inducers p53, caspase 3 and NF*_k_*B vital to apoptosis were further analyzed using the ELISA assay to examine the enantioselectivity at the protein transcription level. Data in **Figure 5** implied that *rac-o,p*’-DDT and two enantiomers had the ability to induce the transcription of proteins (*p*<0.05). However, there was a significant difference between the two enantiomers in their potential for upregulating apoptotic proteins (*p*<0.05). The average level of p53 and caspase 3 exposed to *R*-(−)-*o,p*’-DDT was 1.8- and 1.6-fold higher than that exposed to the *S*-form. The most noticeable enantioselective induction was observed on NF*_k_*B protein when *R*-(−)-*o,p*’-DDT induced as much as 2-fold as compared to the other enantiomer. In summary, *o,p*’-DDT enantioselectively induced apoptotic proteins in the order of *R*-(−)-*o,p*’-DDT > S-(+)-*o,p*’-DDT.

## Discussion

### Environmental Significance of Chiral DDT and its Implications

Decades of research on DDT toxicity often neglected cytotoxicity, whereas chirality of DDT has been largely ignored in risk assessment associated with its use during the last 70 years. Only limited data are available suggesting DDT isomers were apoptosis inducers in several types of cell lines [30]–[32]. In the present study using an array of toxicity tests, we demonstrated how *R*-(−)-*o,p*’-DDT induced more cellular toxicity than *S*-(+)-*o,p*’-DDT under the concentration typical of malaria control area. Translating this finding into a practical avenue for health risk assessment of DDT is, however, not a straightforward task. Partially, this is because the environmental significance for any chiral pesticide is dependent of both degradation and toxicity [18]. For example, if two enantiomers have the same degradation or transformation but different enantioselective toxicity, the enantiomer ratio will remain the same over time and the risk will be predictable from the racemate. Since enantioselective degradation in human and other vertebrates [33], enantiomeric profiling in environmental soils and sediments [34], and stereospecific cytotoxicity in mammalian cells and organs [20]–[23], [35], [36] have been frequently reported, the environmental significance of enantioselectivity of *o,p*’-DDT is warranted.

Questions further remain, however, as how to estimate the enantioselective behavior in a predictable manner and how to incorporate such data into risk assessment. In our previous study with fenamiphos, a consistently higher toxicity to daphnid was obtained for *R*- than *S*-fenamiphos. Using a computer-aided molecular docking simulation tool widely applied in drug designs, we determined that the three-dimensional (3-D) chemical structure of *R*-fenamiphos favors its hydrogen bonding with the active site of the acetylcholinesterase. Unfortunately, this simulation approach cannot be readily applied to distinguish the enantioselectivities of *o,p*’-DDT because the evaluation must consider the 3-D structure of the chiral pesticide, but also the chiral environment of the unknown biological receptor [37].

### Enantioselectivity as Measured by Oxidative Stress Biomarkers

**Figure 6:**
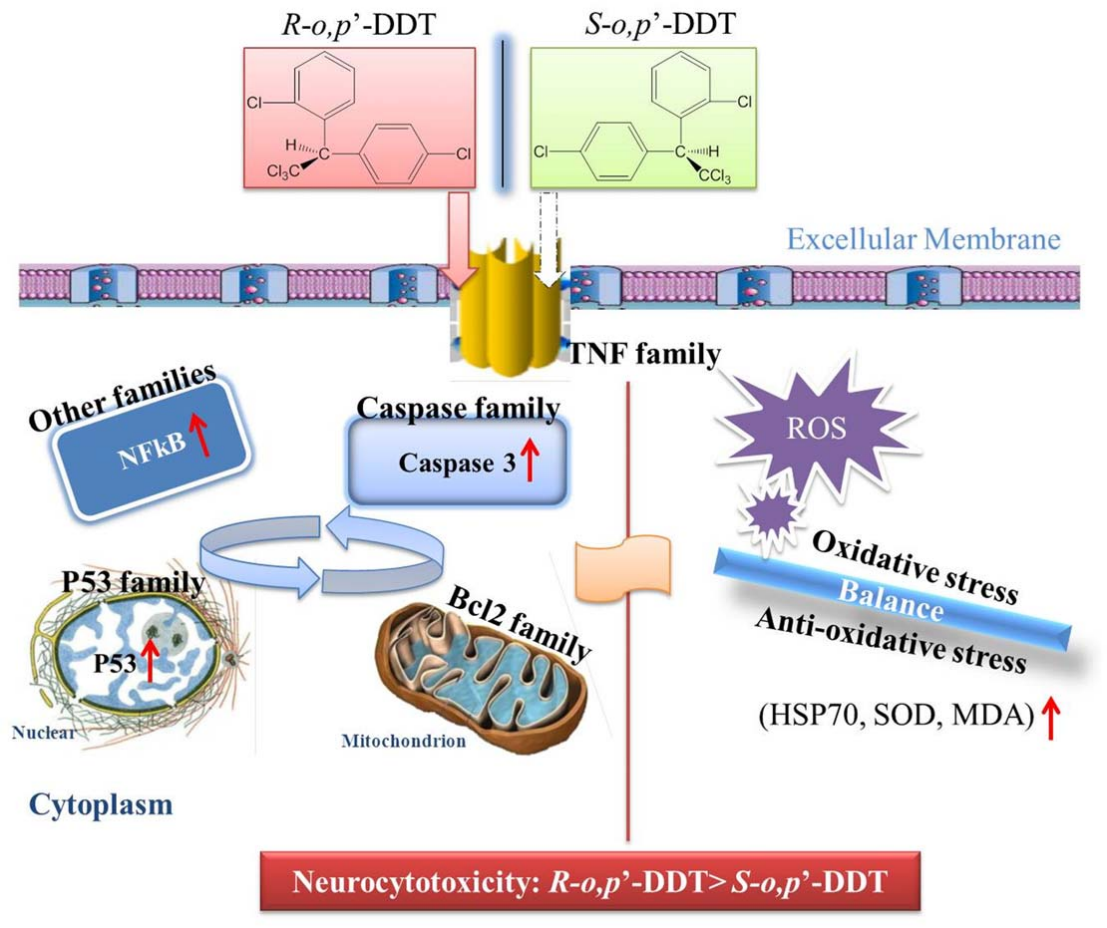
A speculated signaling pathway mediated by *o,p*’-DDT for the induction of cells apoptosis. The red arrowheads indicate the more significant upregulation of apoptosis-related molecules induced by *R*-form than that of *S*-form. Reactive oxygen species (ROS), Heat shock proteins (HSPs), Lactate dehydrogenase (LDH), Superoxide dismultase (SOD), Malondialdehyde (MDA).

Our work is the first reported effort to elucidate the enantioselective effects of DDT on neuron cytotoxicity. With an array of neurocytotoxicity related endpoints, we were able to provide an insightful working model as shown in **Fig. 6**. In this plausible pathway, selective oxidative stress and apoptosis alone or together could contribute to the enantioselective cytotoxicity.

At first, one should note several biomarkers reliable for measuring oxidative stress (**Fig. 6**), including LDH, MDA, SOD, HSPs, SOD1 and SOD2. Chiral pesticides induced enantioselective oxidative stresses have been widely observed for their alteration of SOD enzyme activity, induction of reactive oxygen species (ROS) and MDA, and the decreased chloramphenicol acetyltransferase (CAT) and glutathione (GSH) activity [36], [38]–[42].

One salient feature of observed enantioselectivity is that *R*-form enantioselectively overproduced the level of MDA, more LDH leakage related to cell damage, and significant (*p*<0.05) upregulation of HSP70 associated with the expression of heat shock protein [43]–[45]. This is clearly an indicative of the higher toxicity and stress intensity of PC12 cells following *R*-form treatment. This result is in accordance with the significant (*p*<0.05) increase of SOD induced by *S*-(+)-*o,p*’-DDT, as compared to a decreased SOD by the *R*-form. Since MDA is a reactive oxidant and SOD is an anti-oxidative enzyme, our observations imply the toxic effect under the exposure to *R*-form as opposed to the stress protective effect for its counterpart. Another notable feature is that the mRNA and protein levels do not always correlate in respect to the enantioselectivity of *o,p*’-DDT, since in principle an increased level of oxidant following pesticide treatment will result in the induction of antioxidant genes such as SOD1 and SOD2 [43]. In our study, however, the decreased enzymatic activity of SOD was coupled with the upregulation of SOD1 by *R-*(−)*-o,p*’-DDT, whereas the racemate and *S*-(+)-*o,p*’-DDT increased the SOD activity along with the downregulation of SOD gene transcription. All these results pointed to the fact that *o,p’*-DDT enantioselectively induced cytotoxicity in PC12 cells, which was characterized by a rapid increase in cellular level of toxic oxidant and HSPs along with an imbalance between intracellular antioxidant enzyme and genes expression. The significant response of oxidative stress related genes detected here may shed some light on future research on enantioselective toxicity of chiral chemicals.

### Molecular Mechanism of DDT-induced Apoptosis Leading to Neuron Cytotoxicity

While oxidative stress is a good inducer of apoptosis, cellular apoptosis can exert direct neuron toxicity through the stimulation by a series of orderly signal cascades. Up to now, only mitogen-activated protein kinase (MAPK) pathway has been studied on DDT induced cellular apoptosis [44], [45]. Our group was able to show enantioselective regulation of molecular p53 and Bcl-2 in response to other chiral pesticides [21], [35], [46], however, we were unable to identify any prior studies regarding the possible molecular mechanisms of DDT-induced enantioselective apoptosis.

Cell apoptosis can be mediated by genes in several families. First, as an extracellular mediator, TNF family is important in DDT-induced apoptosis because of its ability to activate some pro-apoptoptic proteins (e.g., caspase) or inhibiting some anti-apoptopic proteins (e.g., Bcl-2). Both *o,p*’- and *p,p*’-DDT were reported to increase TNFα mRNA level and stimulate the TNFα promoter in human embryonic kidney cells, which ultimately induced cell death [45]. In this study, we also observed the changes in the expression of TNF superfamily. A better attenuated expression of TNF family such as Faslg, TNFα, CD40lg genes by *S*-enantiomer seems to partly account for the observed enantioselective cytotoxicity. TNF superfamily causes recruitment of several intracellular adaptors to activate multiple signal transduction pathways, such as apoptosis, NF*_k_*B pathway, and JNK pathway [47].

Following alterations of TNF and its superfamily, the activation of caspase becomes the central event of the apoptosis. The present data showed that caspase-1 and -8 vary in the same direction by both enantiomers (both increase caspase-1 and both decrease caspase-8), whereas an induction of caspase 8 was not observed (Table S3). The vulnerable and critical caspase 3 is commonly observed in many pesticide-induced apoptosis [45], [48]. Consistent with genetic transcription, the higher protein level of caspase 3 induced by *R*-(−)-*o,p*’-DDT reconfirmed its contributing role in the enantioselective toxicity induction.

The Bcl-2 family governs mitochondrial outer membrane permeabilization and can exert either pro- or anti-apoptoptic effect. The overexpression of pro-apoptotic genes of Bcl-2 family have been observed in neuron cell apoptosis [49]–[51]. In our study, *o,p’*-DDT mainly upregulated six pro-apoptotic genes (Bax, Bag, Bcl2-l11, Bid, Bik and Bnip3). An excess of pro-apoptotic proteins can make the cells more sensitive to apoptosis, thereby amplifying cell apoptosis. The more induction of pro-apoptotic genes on Bcl2l11, Bid3 and Bnip3 by *R*-(−)-*o,p*’-DDT may partly explain the higher potency of *R*-(−)-*o,p’*-DDT in causing cell apoptosis.

Concerning the signaling cascade after the alterations of caspase and Bcl-2 members, one can further notice cell physiological changes such as DNA fragmentation and chromatin condensation characterized by p53 family induction. *o,p’*-DDT treated cells increased the Tp53, Trp53bp2 and Gadd45a, as well as Trp63, Trp73 mRNA expression. The activation of Tp53 may be commonplace in neurotoxicity. After treating neural cells with paraquat, the mRNA levels of Tp53, Tp53bp2 and Gadd45a were all upregulated [52]. The expression of Tp53 showed enantioselective activity under *o,p*’-DDT treatment. The protein level of p53 was higher following exposure to *R*-(−)-*o,p*’-DDT than *S*-(+)-*o,p*’-DDT, which was opposite to the gene transcription. This enantioselective upregulation of p53 gene expression was also observed in response to insecticide acetofenate [36]. Finally, the observed upregulation of NF*_k_*B protein indicated that an activation of NF*_k_*B pathway might also be involved in the enantioselectively induced apoptosis by *o,p*’-DDT enantiomers. In conclusion, the enantioselective apoptosis might involve three signaling pathways via caspase 3, tumor protein 53 (p53) and NF*_k_*B.

In summary, since DDT usage in IRS will be prevalent in the foreseeable future, the relative higher dose exposure should be concerned especially in malaria control countries. The acquisition of enantioselective modes of action, casual relationships among observed toxicological endpoints, and quantitative dose-response data will be essential to help us establish more accurate environment standards needed for risk assessment. The shortcoming of current values of slope factor and reference dose of DDT set forth by the U.S. EPA without consideration of enantiomers is evident, and an appropriate toxicity model [53] should be examined for *o,p’*-DDT and any other chiral environmental contaminants.

## Materials and Methods

### Chemical Reagents and Culture Medium

The analytical standard of racemic *o,p*’-DDT [98.5%, 1,1,1-trichloro-2-(o– chlorophenyl)-2-(*p*-chlorophenyl) ethane] was purchased from Bestown (Beijing, China). Stock solutions (10^−2^ mol/L) of *o,p*’-DDT were prepared in ethanol. Dulbecco’s modified Eagle’s medium (DMEM)/F12 and fetal bovine serum (FBS) (Hyclone, Logan, UT, USA) were purchased from Katimesbio (Hangzhou, China). The reagent kits for lactate dehydrogenase (LDH), superoxide dismutase (SOD) and malondialdehyde (MDA) were obtained from Nanjing Jiancheng Bioengineering Institute (China). Solvents for enantiomer separation were all high-performance liquid chromatography (HPLC) grade which meets ACS specifications (SK Chemicals, China).

### *o,p*’-DDT Enantiomers Separation and Quantitative Analysis

The enantiomers of *o,p*’-DDT were resolved on a Jasco LC-2000 HPLC system (Easton, MD, USA) with Chiralcel OJ-H column (0.46 cm internal diameter ×25 cm; Daicel Chemical Industries, Tokyo, Japan). The chromatographic condition of 100% n-hexane at 0.4 mL/min was similar to our previous report [19]. Concentrations of manually collected eluent were determined using an Agilent 6890N gas chromatograph equipped with an electron capture detector (Wilmington, DE, USA).

### Cell Culture and *o,p*’-DDT Treatment

The rat pheochromocytoma 12 cell line was obtained from the cell bank at the Chinese Academy of Sciences. They were maintained at 37°C with 5% CO_2_ in DMEM/F12 containing 10% FBS. PC12 were seeded in culture plates (Coaster) at a density of 1×10^5^ cells/mL. After adhering to plates, the experimental medium (containing 5% FBS) with or without pesticides was added for a period of 24 h. Cells and experimental medium were then collected for testing with ethanol (<0.4%) served as the negative control. All experiments were performed within 30 passages. The exposure doses were set at 1×10^−5^, 2×10^−5^, 3.5×10^−5^ mol/L for *o,p*’-DDT affected enzymatic activity measurement. Based on the results (Figure S1 and Figure S2) from racemate and MTT assay (a colorimetric cell proliferation test), the effect threshold for enantiomer treatment was set at 3.5×10^−5^ mol/L in the following assays.

### Measurement of LDH Release and Intracellular SOD and MDA

LDH is an oxidoreductase released into tissues or extracellular environment when cells are damaged. SOD is an important antioxidant enzyme that produces hydrogen peroxide and molecular oxygen. MDA, a reactive aldehyde-type oxidant, is regarded as a reliable biomarker in lipid peroxidation [54]. These three indexes were determined spectrophotometrically with an assay kit according to a manufacturer’s protocol (see Supporting Information). Together, they were used to measure oxidative stress triggered by *o,p*’-DDT.

### Determination of Cell Apoptosis by FACS

Apoptosis, another inducer of cytotoxicity, is a common endpoint in organisms when exposed to environmental pollutants. The cellular apoptosis was determined using Annexin-V FITC/PI double staining apoptosis kit (Multisciences, China) with a flow cytometer (Becton Dicklin Lakes, NJ, USA) (see Supporting Information).

### RT-PCR for Antioxidative Gene Investigation and Microarray Verification

Quantitative real-time polymerization chain reaction (qRT-PCR) was performed to quantify the expression of antioxidative genes (Table S1) and validate the results obtained from microarray assay. The detailed procedures can be found in Supporting Information.

### PC12 Cells Apoptosis PCR Array

Microarray experiments allow for a comparison of gene expression profiles between treatment and control mRNA samples in a single database. It also allows gene expression profiles from different samples to be compared with each other and analyzed together. PCR-microarray for 84-apoptotic related genes was performed in an effort to provide a molecular mechanism for DDT-induced apoptosis using the rat Apoptosis RT^2^ Profiler™ PCR Array. The array includes the tumor necrosis factor (TNF) ligands and their receptors, members of the B-cell lymphoma 2 (Bcl2, anti-apoptotic/pro-apoptotic proteins), cysteine-aspartic acid protease (caspases), inhibitor of apoptotic (IAP), TNF receptor-associated factor (TRAF), caspase recruitment domain (CARD), death domain, death effectors domain, cell death-inducing DNA fragmentation factor-α-like effector (CIDE) families, and genes involved in the DNA damage. Transcripts with a ratio of change >1.4 or <0.7 were delineated as upregulated or downregulated, respectively [55].

### The Protein Expression of Caspase 3, p53, NFkB by ELISA Assay

The protein levels of tumor protein 53 (p53), cystein-aspartic acid protease 3 (caspase 3) and nuclear factor kappa-light-chain-enhancer of activated B cells (NF*_k_*B) were assessed from *o,p*’-DDT treated cells by an enzyme-linked immunosorbent assay (ELISA). Cells were allowed to attach in 35 cm^2^ dishes overnight and exposed to test chemicals. After 24 h treatment, the whole cell protein was extracted according to a nuclear extract kit (Active Motif North America, Catalog # 40010). The amount of protein expressed was assayed by the ELISA kits (R&D System, China). The concentrations of p53, caspase 3 and NFkB were determined from their respective standard curves obtained under the same conditions as samples. All concentrations of p53, caspase 3 and NFkB were expressed as the mass of the target molecules per µg protein.

### Statistical Analysis

Results are shown as mean ±SD with at least 3 independent replicates. The statistical significance of the differences between two groups was determined by Student’s t test and the multiple comparison among two enantiomers and the racemate was tested by ANOVA, with a probability value of *p*<0.05 considered statistically significant.

### Associated Content

#### Supporting information

One figure for cell proliferation test results, one table for the primers of anti-oxidative related genes, plus ten tables of microarray results are listed in Supporting Information. Additional experimental details are also provided in Supporting Information.

## Supporting Information

Figure S1 Cell proliferation test results for *rac*-*o,p*’-DDT (A) and two enantiomers of *o,p*’-DDT (B).
(TIF)

Figure S2 The oxidative stress (endpoint: SOD, MDA and LDH) for *rac*-*o,p*’-DDT in two concentrations.
(TIF)

Table S1 Gene names and GeneBank accession numbers for stress-related genes
(DOCX)

Table S2 The relative fold change of TNF ligand and receptor family
(DOCX)

Table S3 The relative fold change of caspase family
(DOCX)

Table S4 The relative fold change of Bcl2 family
(DOCX)

Table S5 The relative fold change of p53 and DNA-damage induced apoptosis family
(DOCX)

Table S6 The relative fold change of other genes
(DOCX)

Table S7 The relative fold change of IAP family
(DOCX)

Table S8 The relative fold change of TRAF family
(DOCX)

Table S9 The relative fold change of CARD family
(DOCX)

Table S10 The relative fold change of CIDE domain family
(DOCX)

Table S11 The relative fold change of death domain and receptor domain family
(DOCX)

Supporting Information S1 Additional experimental details.
(DOC)
