# Supplementary figures and images for: Enantioselective Cytotoxicity Profile of o,p’-DDT in PC 12 Cells
Source: PLoS One. 2012 Aug 24;7(8):e43823. doi: 10.1371/journal.pone.0043823 (PMC3427172; doi:10.1371/journal.pone.0043823)

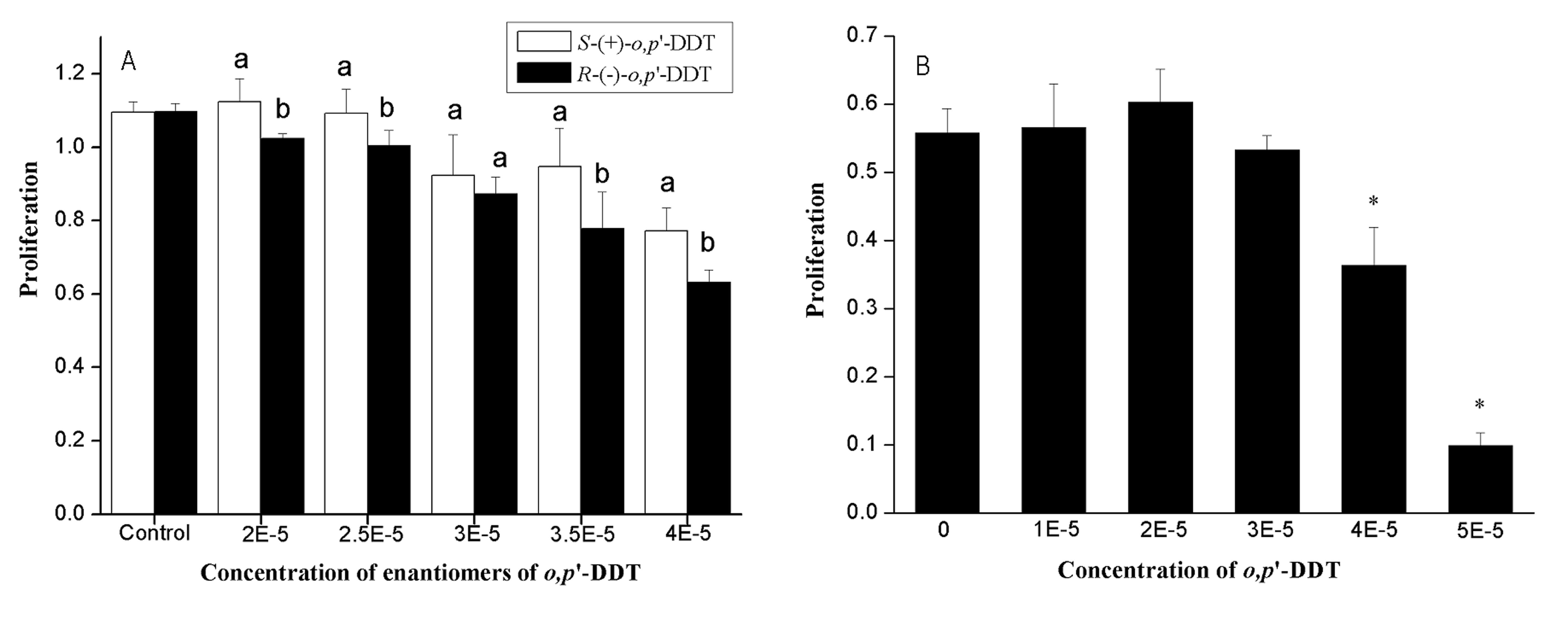

Supplement: Figure S1 — Cell proliferation test results for rac-o,p’-DDT (A) and two enantiomers of o,p’-DDT (B). (TIF) [file pone.0043823.s001.tif]
