# Supplementary material for: Enantioselective Cytotoxicity Profile of o,p’-DDT in PC 12 Cells
Source: PLoS One. 2012 Aug 24;7(8):e43823. doi: 10.1371/journal.pone.0043823 (PMC3427172; doi:10.1371/journal.pone.0043823)
Supplement: Table S1 — Gene names and GeneBank accession numbers for stress-related genes (DOCX) [file pone.0043823.s003.docx]

Table S1. Gene names and GeneBank accession numbers for stress-related genes

| Gene  symbol | Gene name | Sequence | GeneBank  accession no. |
| --- | --- | --- | --- |
| sod1 | superoxide dismutase 1 | 3’-CGGATGAAGAGAGGCATGTT  5’- CACCTTTGCCCAAGTCATCT | NM_017050.1 |
| sod2 | superoxide dismutase 2 | 3’-CCGAGGAGAAGTACCACGAG  5’-GCTTGATAGCCTCCAGCAAC | NM_017051.2 |
| hsp70 | heat shock protein, 70 | 3’-CAGGATTTGCCCTATCCAGA  5’-CTCTACTAAGGCCGCACTGG | NM_153629.1 |
