# Supplementary material for: Enantioselective Cytotoxicity Profile of o,p’-DDT in PC 12 Cells
Source: PLoS One. 2012 Aug 24;7(8):e43823. doi: 10.1371/journal.pone.0043823 (PMC3427172; doi:10.1371/journal.pone.0043823)
Supplement: Table S2 — The relative fold change of TNF ligand and receptor family (DOCX) [file pone.0043823.s004.docx]

Table S2.The relative fold change of TNF ligand and receptor family

| Gene names | *Rac*-*o,p*’-DDT | *S*-(+)-*o,p’*-DDT | *R*-(-)-*o,p*’-DDT | S/R |
| --- | --- | --- | --- | --- |
| Lta | 1.4 | -2.5 | -2.0 | 0.85 |
| Tnf | 1.4 | -2.0 | -1.1 | 0.55 (1.82) |
| Tnfsf10 | 1.7 | -2.0 | -1.4 | 0.84 |
| Tnfsf12 | 1.4 | 1 | -1.1 | 1.09 |
| Cd40lg | 1.0 | -2.5 | -2.0 | 0.8 (1.25) |
| Faslg | 1.6 | -2.0 | -1.4 | 0.69 (1.43) |
| Ltbr | 1.9 | 1.0 | -1.4 | 1.34 |
| Tnfrsf10b | 1.1 | -2.5 | -2.5 | 0.88 |
| Tnfrsf11b | 2.0 | -2.0 | -1.7 | 0.91 |
| Tnfrsf1a | 2.1 | 1.2 | 1.2 | 0.95 |
| Tnfrsf1b | 2.5 | 1.5 | 1.5 | 0.96 |
| Tnfrsf5 | 1.7 | -1.7 | -1.4 | 0.80 |
| Tnfrsf6 | 1.7 | -1.7 | -1.7 | 0.98 |
