# Supplementary material for: Enantioselective Cytotoxicity Profile of o,p’-DDT in PC 12 Cells
Source: PLoS One. 2012 Aug 24;7(8):e43823. doi: 10.1371/journal.pone.0043823 (PMC3427172; doi:10.1371/journal.pone.0043823)
Supplement: Table S3 — The relative fold change of caspase family (DOCX) [file pone.0043823.s005.docx]

Table S3.The relative fold change of caspase family

| Gene names | *Rac*-*o,p*’-DDT | *S*-(+)-*o,p’*-DDT | *R*-(-)-*o,p*’-DDT | S/R |
| --- | --- | --- | --- | --- |
| Caspase1 | 1.0 | -2.5 | -1.25 | 0.44 (2.27) |
| Caspase 4 | 7.3 | 1.2 | 1.2 | 1.06 |
| Caspase 12 | 2.7 | 1.1 | 1.1 | 1.03 |
| Caspase 14 | 1.2 | -1.4 | -1.1 | 0.73 |
| Caspase 2 | 2.0 | 1.4 | 1.3 | 1.08 |
| Caspase 3 | 1.9 | 1.7 | 2.6 | 0.62(1.61) |
| Caspase 6 | 1.2 | -1.4 | 1.0 | 0.77 |
| Caspase 7 | 1.8 | -1.1 | -1.25 | 1.10 |
| Caspase 8 | 1.4 | -1.25 | -1.1 | 0.89 |
| Caspase 8ap2 | -2.0 | -2.5 | 1.8 | 0.22(4.55) |
| Caspase 9 | 1.6 | 1.0 | -1.1 | 1.13 |
