# Supplementary material for: Enantioselective Cytotoxicity Profile of o,p’-DDT in PC 12 Cells
Source: PLoS One. 2012 Aug 24;7(8):e43823. doi: 10.1371/journal.pone.0043823 (PMC3427172; doi:10.1371/journal.pone.0043823)
Supplement: Table S4 — The relative fold change of Bcl2 family (DOCX) [file pone.0043823.s006.docx]

Table S4.The relative fold change of Bcl2 family

| Gene names | *Rac*-*o,p*’-DDT | *S*-(+)-*o,p’*-DDT | *R*-(-)-*o,p*’-DDT | S/R |
| --- | --- | --- | --- | --- |
| Bad | -2.0 | -1.25 | 1.0 | 0.78 |
| Bid | 2.3 | -1.1 | -1.1 | 0.92 |
| Bag1 | 1.5 | 1.1 | -1.1 | 1.13 |
| Bak1 | 1.2 | 1.0 | 1.0 | 0.94 |
| Bax | 1.9 | -1.1 | 1.0 | 0.89 |
| Bcl10 | .0.6 | 0.8 | 1.0 | 0.78 |
| Bcl2 | 2.1 | 1.0 | 1.0 | 1.03 |
| Bcl2a1 | 1.9 | -1.7 | -1.4 | 0.88 |
| Bcl2l1 | 1.1 | -1.7 | -1.1 | 0.68(1.47) |
| Bcl2l2 | 1.6 | 1.0 | -1.1 | 1.13 |
| Bcl2l11 | 1.6 | -1.4 | 1.6 | 0.46(2.17) |
| Bclaf1 | 1.3 | -1.1 | -1.25 | 1.11 |
| Bid3 | 1.0 | -2.5 | -1.25 | 0.54(1.85) |
| Bik | 1.6 | -2.0 | -1.7 | 0.85 |
| Bnip1 | 1.2 | -1.25 | -1.4 | 1.21 |
| Bnip2 | 1.1 | -1.25 | -1.25 | 1.00 |
| Bnip3 | 1.6 | -1.1 | 2.1 | 0.43(2.33) |
| Mcl1 | 1.9 | 1.1 | -1.1 | 1.19 |
| Bok | 1.2 | -1.25 | -1.25 | 1.04 |
