# Supplementary material for: Enantioselective Cytotoxicity Profile of o,p’-DDT in PC 12 Cells
Source: PLoS One. 2012 Aug 24;7(8):e43823. doi: 10.1371/journal.pone.0043823 (PMC3427172; doi:10.1371/journal.pone.0043823)
Supplement: Table S5 — The relative fold change of p53 and DNA-damage induced apoptosis family (DOCX) [file pone.0043823.s007.docx]

Table S5.The relative fold change of p53 and DNA-damage induced apoptosis family

| Gene names | *Rac*-*o,p*’-DDT | *S*-(+)-*o,p’*-DDT | *R*-(-)-*o,p*’-DDT | S/R |
| --- | --- | --- | --- | --- |
| Gadd45a | 2.9 | -1.4 | 1.2 | 0.61(1.64) |
| p53 | 2.3 | 1.7 | 1.1 | 1.52 |
| Trp53bp2 | 1.6 | -1.25 | -1.1 | 0.91 |
| Trp63 | 1.6 | -2.0 | -1.4 | 0.65(1.4) |
| Trp73 | 1.5 | -2.0 | -1.4 | 0.76 |
