# Supplementary material for: Enantioselective Cytotoxicity Profile of o,p’-DDT in PC 12 Cells
Source: PLoS One. 2012 Aug 24;7(8):e43823. doi: 10.1371/journal.pone.0043823 (PMC3427172; doi:10.1371/journal.pone.0043823)
Supplement: Table S6 — The relative fold change of other genes (DOCX) [file pone.0043823.s008.docx]

Table S6.The relative fold change of other genes

| Gene names | *Rac*-*o,p*’-DDT | *S*-(+)-*o,p’*-DDT | *R*-(-)-*o,p*’-DDT | S/R |
| --- | --- | --- | --- | --- |
| Api5 | 1.0 | -1.4 | -1.1 | 0.80 |
| Aven | 1.4 | -1.4 | 1.1 | 0.62(1.61) |
| Dad1 | 1.1 | 1.0 | 1.2 | 0.77 |
| Faim | -1.7 | -2.0 | -1.4 | 0.68(1.47) |
| Il10 | 1.2 | -2.0 | -1.7 | 0.90 |
| Lhx4 | 1.2 | -2.5 | -2.0 | 0.81 |
| Mapk8ip | 2.0 | 1.3 | 1.6 | 0.84 |
| NFkb1 | 1.8 | 1.0 | 2.4 | 0.43(2.35) |
| Polb | 1.5 | -1.25 | 1.0 | 0.73 |
| Prlr | 1.7 | -1.7 | -1.4 | 0.88 |
| Prdx2 | -1.4 | -1.7 | -1.25 | 0.81 |
| Prok2 | 1.2 | -2.0 | -1.7 | 0.73 |
| Sphk2 | -1.4 | -2.5 | -2.0 | 0.81 |
