# Supplementary material for: Enantioselective Cytotoxicity Profile of o,p’-DDT in PC 12 Cells
Source: PLoS One. 2012 Aug 24;7(8):e43823. doi: 10.1371/journal.pone.0043823 (PMC3427172; doi:10.1371/journal.pone.0043823)
Supplement: Table S7 — The relative fold change of IAP family (DOCX) [file pone.0043823.s009.docx]

Table S7.The relative fold change of IAP family

| Gene names | *Rac*-*o,p*’-DDT | *S*-(+)-*o,p’*-DDT | *R*-(-)-*o,p*’-DDT | S/R |
| --- | --- | --- | --- | --- |
| Birc1b | 1.3 | -2.0 | -1.25 | 0.61(1.64) |
| Birc3 | 1.3 | -1.7 | 1.0 | 0.62(1.61) |
| Birc4 | 1.2 | -1.25 | 1.2 | 0.69(1.45) |
| Birc5 | 1.1 | -1.1 | -1.1 | 1.07 |
