# Supplementary material for: Enantioselective Cytotoxicity Profile of o,p’-DDT in PC 12 Cells
Source: PLoS One. 2012 Aug 24;7(8):e43823. doi: 10.1371/journal.pone.0043823 (PMC3427172; doi:10.1371/journal.pone.0043823)
Supplement: Table S8 — The relative fold change of TRAF family (DOCX) [file pone.0043823.s010.docx]

Table S8.The relative fold change of TRAF family

| Gene names | *Rac*-*o,p*’-DDT | *S*-(+)-*o,p’*-DDT | *R*-(-)-*o,p*’-DDT | S/R |
| --- | --- | --- | --- | --- |
| Traf1 | 1.1 | -2.0 | -1.4 | 0.74 |
| Traf2 | 1.5 | -1.25 | 1.0 | 0.84 |
| Traf3 | 1.6 | 1.0 | 1.0 | 0.97 |
| Traf4 | 2.4 | 1.3 | 1.2 | 1.16 |
