# Supplementary material for: Enantioselective Cytotoxicity Profile of o,p’-DDT in PC 12 Cells
Source: PLoS One. 2012 Aug 24;7(8):e43823. doi: 10.1371/journal.pone.0043823 (PMC3427172; doi:10.1371/journal.pone.0043823)
Supplement: Table S9 — The relative fold change of CARD family (DOCX) [file pone.0043823.s011.docx]

Table S9.The relative fold change of CARD family

| Gene names | *Rac*-*o,p*’-DDT | *S*-(+)-*o,p’*-DDT | *R*-(-)-*o,p*’-DDT | S/R |
| --- | --- | --- | --- | --- |
| Apaf1 | 1.0 | -1.1 | -1.4 | 0.81 |
| Card6 | 1.0 | -1.7 | -1.25 | 0.80 |
| Card10 | 1.4 | -1.7 | -1.7 | 0.87 |
| Cradd | 1.3 | -1.4 | -1.4 | 1.04 |
| Nol3 | 4.8 | 1.2 | 1.7 | 0.72 |
| Pycard | 1.2 | -1.4 | -1.25 | 0.95 |
| Ripk2 | 1.5 | -1.1 | -1.25 | 1.06 |
