# Supplementary material for: Enantioselective Cytotoxicity Profile of o,p’-DDT in PC 12 Cells
Source: PLoS One. 2012 Aug 24;7(8):e43823. doi: 10.1371/journal.pone.0043823 (PMC3427172; doi:10.1371/journal.pone.0043823)
Supplement: Table S11 — The relative fold change of death domain and receptor domain family (DOCX) [file pone.0043823.s013.docx]

Table S11.The relative fold change of death domain and receptor domain family

| Gene names | *Rac*-*o,p*’-DDT | *S*-(+)-*o,p’*-DDT | *R*-(-)-*o,p*’-DDT | S/R |
| --- | --- | --- | --- | --- |
| Dapk1 | 1.8 | -1.4 | -1.4 | 1.02 |
| Fadd | 1.4 | -1.7 | -1.1 | 0.69(1.45) |
| Tradd | 1.6 | -1.1 | 1.0 | 0.91 |
| Cflar | 1.3 | -1.1 | 1.0 | 0.81 |
