# Supplementary material for: Enantioselective Cytotoxicity Profile of o,p’-DDT in PC 12 Cells
Source: PLoS One. 2012 Aug 24;7(8):e43823. doi: 10.1371/journal.pone.0043823 (PMC3427172; doi:10.1371/journal.pone.0043823)
Supplement: Supporting Information S1 — Additional experimental details. (DOC) [file pone.0043823.s014.doc]

*Supporting Information*

**Enantioselective Cytotoxicity Profile of *o,p*’-DDT**

**in PC 12 Cells**

Meirong Zhao,**^a^** Cui Wang,**^a^**

*Research Center of Environmental Science, Zhejiang University of Technology, Hangzhou 310032, China*

Chunlong Zhang

*University of Houston-Clear Lake, Houston, TX 77058, USA*

Yuezhong Wen, Weiping Liu^*^

*Institute of Environmental Sciences, College of Environmental and Resource Sciences, Zhejiang University, Hangzhou 310058, China*

^a^The authors make equal contribution to the paper.

**S2. Materials and Methods**

**S2.1 Determination of cell apoptosis by FACS**

PC12 were cultured in 35 mm dishes with or without pesticides for 24h and then typsinized, washed, and re-suspend in 500μL of 1× binding buffer. This was followed by the addition of 5μLof Annexin V-FITC and 10μL of propidium iodide (PI) and the incubation at room temperature for 5 min in the dark. Samples were then analyzed by a flow cytometer (Becton Dickinson, Franklin Lakes, NJ, USA). Results were obtained from four independent replicates.

**S2.2 RT-PCR for antioxidative gene investigation and microarray verification**

Total RNA (1μg) was reverse transcribed into cDNA (ReverTra Ace qPCR RT kit) following the manufacturer’s instructions. Q-PCR protocol included the following: diluted cDNA sample (1:10 (v/v)), 5×RT buffer, RT enzyme mix, primers mix, and buffer included in the SYBR Green PCR master mix (Toyobo).Using the 7300real-time PCR system (Applied Biosystems, Foster City, CA, USA). PCR cycling conditions were as follows: 95 ºC for 1 min, followed by 40 cycles at 95 ºC for 15 seconds, and 60 ºC for 1 min and the melting curve analyses were set at 95 ºC for 1 min and 65 ºC for 2min.The relative quantification of gene expression among the treatment groups was analyzed by the 2^-ΔΔC^_T_ method.^1^ Each mRNA level was expressed as its ratio to *β-*actin mRNA. Specific oligonucleotide primers for antioxidative genes, apoptotic genes and housekeeping *β*-actin were designed via primer 3.0 software (Oxford Molecular Ltd, Madison, WI, USA) and are listed in **Table S1.**

**S2.3 PC12 cells apoptosis PCR array**

Total RNA was extracted from the three mixed independent dishes of PC12 cells, which was treated byone chemical, using high-purity Rneasy Mini kits (Qiagen, Catalog #74104). The real-time PCR for rat apoptosis microarray assay was performed using the RT^2^ Profiler PCR microarray kit (SuperArray Bioscience, Frederick, MD, USA) in a 96-well format according to the manufacturer’s protocol. In brief, for the reverse transcription, 8 μL of each sample (containing 1μg mRNA) were mixed with 2 μL of GE (5 gDNA Elimination Buffer) to make a total volume of 10μLgenomic DNA elimination mixture. The mixture was denatured at 42 ºC for 5 min and chilled on ice immediately for at least 1 min. Samples were then mixed with 10μL RT cocktail (SuperArray Bioscience RT2 first strand kit C-03), 20μL of RNAse inhibitor for cDNA synthesis performed for exactly 15 min at 42 ºC followed by an enzyme inactivation step of 5 min at 95 ºC. The total volume of the reaction (20μL) was dilluted to 91μL. PCR reactions were performed using Eppendorf Mastercyler ep realplex 4S, with the ramp rate at 26%. The thermo cycler parameters were 95 ºC for 10 min, followed by 40 cycles of 95 ºC for 15 seconds and 60 ºC for 1 min. Among five housekeeping genes glyceraldehydes-3-phosphate dehydrogenase (GAPDH) was used for normalization between samples and relative gene changes in gene expression were analyzed with ΔΔCt method.

**S3. Results**

**S 3.1 Notes on the influence of enantiomers of *o,p*’-DDT on TRAF, CIDE, IAP family**

The high transcriptional level of death domain TRADD, FADD, and Dapk1 under the treatment of DDT underscored the idea that the activated TNF binds with the TRADD, and then recruit FADD. Dapk1 mRNA overexpressed by *o,p*’-DDT might act as a positive regulator of apoptosis through calcium/calmodulin-dependent serine/threonine kinase. The markedly enantioselective regulation of DDT occurred on FADD gene. *S*-(+)-*o,p*’-DDT downregulated FADD to 0.6-fold compared to 0.94-fold induced by *R*-(–)-*o,p*’-DDT. Studies on neuron cells reported a downregulation of FADD in paraquat-induced SH-SY5Y52. Nevertheless, FADD-immunoreactive pathway was speculated as a contribution to the susceptibility of neurons in PD to TNF-mediated apoptosis54. The importance of upregulation or downregulation of FADD in apoptosis remains unknown.

TRAFs proteins mediate the signal transduction members of the TNFrsf. They had been reported significantly increased in paraquat treated neuro blastome cells.^2^ TRAF2 is an activator of NF*_k_*B pathway. Difference in upregulation of NF*_k_*B gene and protein by enantiomers of *o,p*’-DDT in our study indicated that an activation of NF*_k_*B pathway might be involved in the enantioselectiveinduced apoptosis.

**S References**

1. Kedziora-Kornatowska KZ, Luciak M, Paszkowski J (2000) Lipid peroxidation and activtties of antioxidant enzymes in the diabetic kidney: effect of treatment with angiotensin convertase inhibitors. IUBMB life 49: 303-307.
2. Bollimuntha S, Singh BB, Shavali S, Sharma SK, Ebadi M (2005) TRPC1-mediated inhibition of 1-methyl-4-phenylpyridinium ion neurotoxicity in human SH-SY5Y neuroblastoma cells. J Biol Chem 280: 2132–2140.
3. Manufacturer’s instructions
